# Long-term outcome of bioresorbable vascular scaffolds for the treatment of coronary artery disease: a meta-analysis of RCTs

**DOI:** 10.1186/s12872-017-0586-2

**Published:** 2017-06-07

**Authors:** Alberto Polimeni, Remzi Anadol, Thomas Münzel, Ciro Indolfi, Salvatore De Rosa, Tommaso Gori

**Affiliations:** 1 Zentrum für Kardiologie, University Hospital Mainz, Mainz, Germany; 2 German Center for Cardiac and Vascular Research (DZHK), Standort Rhein-Main, Mainz, Germany; 3 Division of Cardiology, Department of Medical and Surgical Sciences, “Magna Graecia” University, 88100 Catanzaro, Italy; 4 URT-CNR, Department of Medicine, Consiglio Nazionale delle Ricerche of IFC, Viale Europa S/N, 88100 Catanzaro, Italy

**Keywords:** Stent thrombosis, Target lesion failure, Bioresorbable vascular scaffold

## Abstract

**Background:**

Coronary bioresorbable scaffolds (BRS) were developed to overcome the limitations of standard metallic stents, especially to address late events after percutaneous coronary interventions. The aim of this meta-analysis was to evaluate the efficacy and safety of BRS, compared with Everolimus-eluting stents (EES), using the data available from randomized trials, with a focus on long-term outcomes.

**Methods:**

Published randomized trials comparing BRS to EES for the treatment of coronary artery disease were searched for within PubMed, Cochrane Library and Scopus electronic databases up to April 4th 2017. The summary measure used was odds ratio (OR) with 95% confidence intervals.

**Results:**

A total of 5 studies were eligible, including 5219 patients. At 2 years, BRS was associated with higher rates of target lesion failure (9.4% vs 7.2%; OR = 1.33; 95% CI 1.07 to 1.63; *p* = 0.008) and device thrombosis (2.3% vs 0.7%; OR = 3.22; 95% CI 1.86 to 5.57; *p* < 0.0001) compared with EES. The incidence of both early (within 30 days after implantation, 1.1% vs 0.5%, OR 1.97, 95% CI 1.02 to 3.81; *p* = 0.05) and very-late device thrombosis (>1 year, 0.6% vs 0.1%, OR 4.03, 95% CI 1.37 to 11.82; *p* = 0.01) was higher with BRS compared with EES.

**Conclusions:**

BRS may be associated with worse two-years clinical outcomes compared with EES in patients with coronary artery disease.

**Electronic supplementary material:**

The online version of this article (doi:10.1186/s12872-017-0586-2) contains supplementary material, which is available to authorized users.

## Background

The introduction of coronary stents has revolutionized interventional cardiology. However, despite significant improvement over the years, traditional metallic stents have some intrinsic limitations. In fact, their permanent structure hinders surgical myocardial revascularization, physiological vessel remodeling and exposes patients to the risk of stent thrombosis for an indefinite time. Coronary bioresorbable scaffolds (BRS) were developed to overcome some of these limitations of standard metallic stents, especially to address late events after percutaneous coronary interventions (PCI) [1]. BRS have been introduced in the last years as a novel promising approach to treat coronary stenosis by providing transient vessel support with drug delivery capability without the long-term limitations associated with vessel caging [2]. This technology has the potential to overcome many of the safety concerns associated with drug-eluting stents, with possible clinical benefits [3]. Although initial reports from single-arm studies in highly selected patients with simple coronary lesions were reassuring [4–6], recent data from “real-life” registries and randomized controlled trials reported that the rates of scaffold-related are not negligible, also at long-term [7–9].

The aim of this meta-analysis was to evaluate the efficacy and safety of BRS, compared with Everolimus-eluting stents (EES), using the data available from randomized trials, with a focus on long-term outcomes.

## Methods

### Search strategy and study selection

Published randomized trials comparing BRS to EES for the treatment of coronary artery disease were searched for within PubMed, Cochrane Library, Scopus electronic databases and scientific sessions abstracts, and relevant websites (www.clinicaltrialresults.org, www.escardio.org, www.tctmd.com) up to April 4th 2017. We checked the reference lists from all eligible studies to identify additional citations. The following keywords and the corresponding MeSH terms were used for search: “bioresorbable vascular scaffold”, “everolimus-eluting stent”, “coronary artery disease”, “randomized controlled trial”. Time of publication and language were not limiting criteria for our analysis. All reports including the search terms were independently screened by two investigators for relevance and eligibility (AP, RA). Additionally, references from relevant articles were also scanned for eligible studies. The authors discussed their evaluation and any disagreement was resolved through discussion and re-reading. All selected trials were thoroughly checked and classified by author’s institution in order to avoid any effect from duplicity of data.

Studies were considered eligible if the following statements were applying: a) they involved a study population with coronary artery disease; b) multicenter randomized controlled trials c) they compared BRS versus EES; d) follow-up length of 2 years; e) they reported outcome data: target lesion failure (TLF), device thrombosis (DvT), cardiac death, target-vessel myocardial infarction (TVMI), ischemia-driven target lesion revascularization (ID-TLR); f) minimum of 100 patients treated with BRS. Exclusion criteria were (just one was sufficient for study exclusion): duplicate publication, pre-specified endpoint, measure not specified. Studies reporting only lesion-based analyses were excluded from the present work.

### Data abstraction, validity assessment and analysis

Baseline characteristics as well as numbers of events were extracted from the single studies, through carefully scanning of the full article by two independent reviewers (AP, SDR). Divergences were resolved by consensus. In particular, the following data were abstracted: year of publication, location, number of study patients, study design, clinical outcome data, baseline patients’ characteristics. Selection and data abstraction was performed according to the PRISMA statement [10]. The primary analysis was based on the composite endpoint of TLF (Cardiac death, TVMI, ID-TLR). Furthermore, meta-analysis results of single endpoints are also provided (Probable/definite DvT; early/late/very late DvT; ischemia-driven target vessel revascularization; target vessel myocardial infarction; cardiac death). Since some of the RCTs pool definite and probable DvT, we evaluated only the incidence of the composite endpoint “definite/probable DvT”.

### Statistical analysis

The summary measure used was the Odds Ratio (OR) with 95% confidence interval. The random-effects model was used, as previously described, to combine the collected values [11]. This model calculates a weighted average of the relative risks by incorporating within-study and between-study variations. Heterogeneity was assessed by means of the Cochrane Q test using a chi-squared function, with *p* values <0·10 considered significant for heterogeneity, as previously described [12]. Additionally, I^2^ values were calculated for estimation of variation in weighted mean differences among studies attributable to heterogeneity. Any I^2^ value >20% was considered significant. Small study effects were evaluated through graphical inspection of funnel plots, as already previously described [13]. Forest plots were used to graphically display the results of the meta-analysis, as already previously described [14]. Briefly, the measure of effect (OR) for each single study included (represented by a square) is plotted, together with confidence intervals, represented by horizontal lines. The area of each square is proportional to the study’s weight in the meta-analysis. The overall measure of effect is reported on the bottom line of the plot as a diamond, whose lateral ends indicate the confidence interval for the summary effect. Analyses were performed by means of RevMan 5.3.

## Results

### Search results

**Fig. 1:**
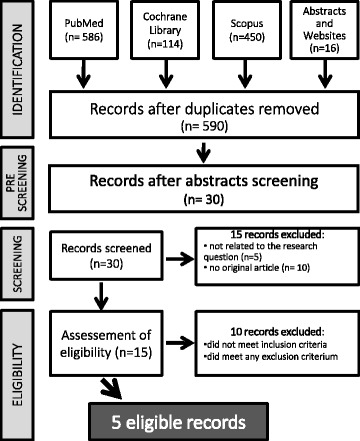
Study selection flow chart

Our search retrieved a total of 590 entries, which were reduced to 30 studies after an initial pre-screening. Fifteen studies were then excluded for one of the following reasons: a) they were not related to our research question b) they weren’t original articles. In the assessment of eligibility further 10 studies were excluded. Finally, a total of 5 studies were available for the analysis including 5219 patients [15–19]. The study selection procedure is reported in detail in Fig. 1.

### Study characteristics

Only multicenter, randomized, trials were included in the present meta-analysis. Table 1 summarizes the most relevant characteristics of the selected studies. Not surprisingly, quality assessment revealed a high study quality (Additional file 1: Figure S1). Moreover, endpoint assessment and data analysis was blinded in all included studies.

**Table 1:** Characteristics and Endpoint definitions of included randomized trials

Study	Year	Location	Number	Study design	Primary endpoint	Definition of TLF	Definition of ST	Follow up (months)	Lost to FU (%)
AIDA	2017	Multicenter	1845	RCT	TVF	Cardiac death, TVMI, ID-TLR	ARC	24	2.8
ABSORB III	2017	Multicenter	2008	RCT	TLF	Cardiac death, TVMI, ID-TLR	ARC	24	2.1
ABSORB China	2016	Multicenter	480	RCT	IS-LL	Cardiac death, TVMI, ID-TLR	ARC	24	3.7
ABSORB II	2016	Multicenter	501	RCT	Vasomotion, MLD	Cardiac death, TVMI, ID-TLR	ARC	24	4.2
ABSORB Japan	2016	Multicenter	400	RCT	TLF	Cardiac death, TVMI, ID-TLR	ARC	24	3

*Abbreviations*: *TLF* target lesion failure, *TVF* target vessel failure, *IS-LL* in-segment late loss, *MLD* minimal lumen diameter, *TVMI* target vessel myocardial infarction, *ID-TLR* ischemia driven target lesion revascularization, *RCT* randomized clinical trials

Across the studies, patients were predominantly male and approximately one fourth of patients had diabetes mellitus. Although prevalence of single cardiovascular risk factors was not equal among the studies, treatment arms were generally well balanced. Dual antiplatelet therapy was used at least 1 year in all the studies. More details on patients’ characteristics are provided in Table 2.

**Table 2:** Baseline patient’s and procedural characteristics

	AIDA 2017		ABSORB III 2017		ABSORB China 2016		ABSORB II 2016		ABSORB Japan 2016	
	BRS	EES	BRS	EES	BRS	EES	BRS	EES	BRS	EES
N of patients, n	924	921	1322	686	238	237	335	166	266	134
Age, yrs.	64	64	63	64	57	58	62	61	67	67
Male, %	72	76	71	70	72	73	76	80	79	74
Hypertension, %	51	50	85	85	59	60	69	72	78	80
Diabetes, %	18	17	31	33	25	23	24	24	36	36
Dyslipidaemia, %	38	38	86	86	42	38	75	80	82	82
Prior MI, %	18	19	21	22	17	16	28	28	16	24
STEMI, %	25		0		0		0		0	
NSTEMI, %	20		0		0		0		0	
UA, %	8		26		64		21		12	
SA, %	40		58		19		64		64	
Silent Ischaemia, %	NR		10		5		12		23	
Intracoronary imaging, %	NR		100	100	0.4	0.4	100	100	100	100
Pre-dilation, %	97	91	100	100	99.6	98	100	99	100	100
Post dilation, %	74	49.1	65.5	51.2	63	54.4	61	59	82.2	77.4

*yrs* years, *MI* myocardial infarction BRS = bioresorbable vascular scaffold, *EES*everolimus-eluting stent, *STEMI* ST-elevation myocardial infarction, *NSTEMI* No ST-elevation myocardial infarction;UA = unstable angina, *SA* stable angina, *NR* not reported

### Meta-analysis results

**Fig. 2:**
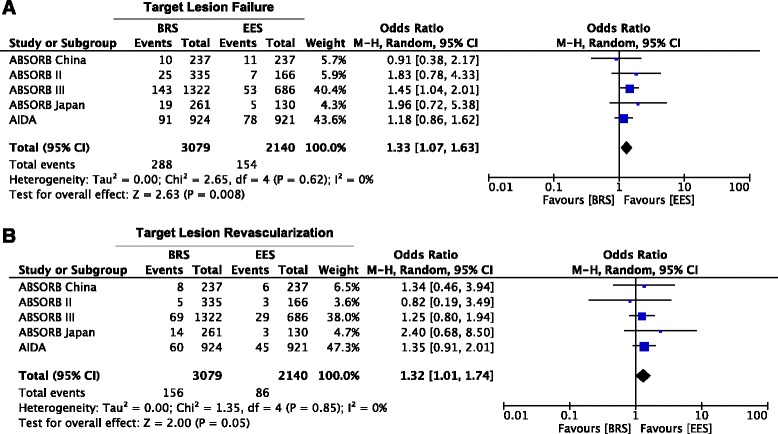
Meta-analysis of Target lesion failure and Target lesion revascularization. Panel **a**. Forest plot and summary effect of the difference in the incidence of TLF, showing a significantly lower incidence in the EES arm (*p* = 0.008). Panel **b**. Forest plot and summary effect of the difference in the incidence of TLR, showing a significantly lower incidence in the EES arm (*p* = 0.05)

**Fig. 3:**
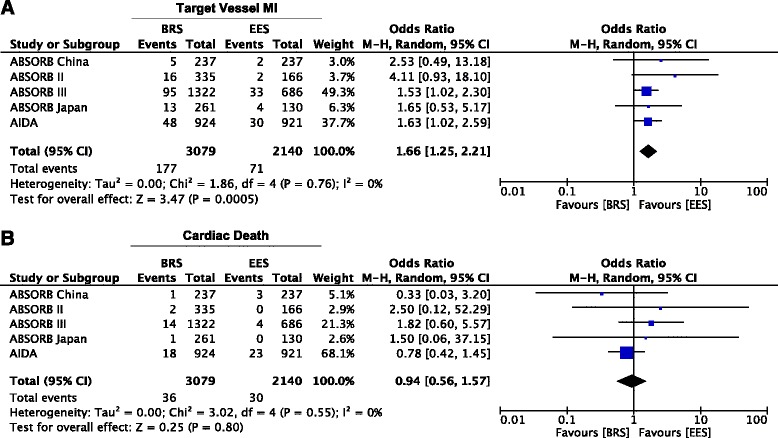
Meta-analysis of Target vessel myocardial infarction and Cardiac Death. Panel **a**. Forest plot and summary effect of the difference in the incidence of TV-MI, showing a significantly lower incidence in the EES arm (*p* = 0.0005). Panel **b**. Forest plot and summary effect of the difference in the incidence of Cardiac death, showing no difference between BRS and EES (*p* = 0.80)

At 2 years, BRS was associated with higher rates of TLF compared with EES (9.4% vs 7.2%; OR = 1.33; 95% CI 1.07 to 1.63; *p* = 0.008, I^2^ = 0%) (Fig. 2a). This result was driven by a major incidence of TLR (5.1% vs 4.0%; OR = 1.32; 95% CI 1.01 to 1.74; *p* = 0.05, I^2^ = 0%) (Fig. 2b) and TV-MI (5.7% vs 3.3%; OR = 1.66; 95% CI 1.25 to 2.21; *p* = 0.0005, I^2^ = 0%)(Fig. 3a) in BRS group compared with EES. No difference in Cardiac death (1.2% vs 1.4%; OR = 0.94; 95% CI 0.56 to 1.57; *p* = 0.80, I^2^ = 0%) was observed between both groups (Fig. 3b).

**Fig. 4:**
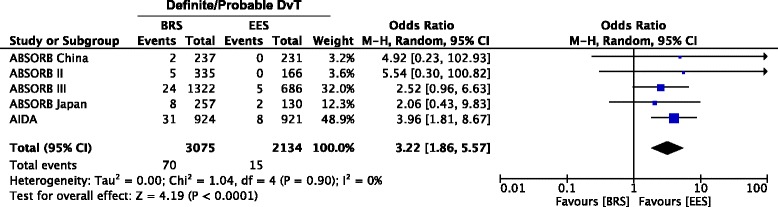
Meta-analysis of Definite/Probable Scaffold Thrombosis. Forest plot and summary effect of the difference in the incidence of Definite/Probable DvT, showing a significantly lower incidence in the EES arm (*p* < 0.0001)

**Fig. 5:**
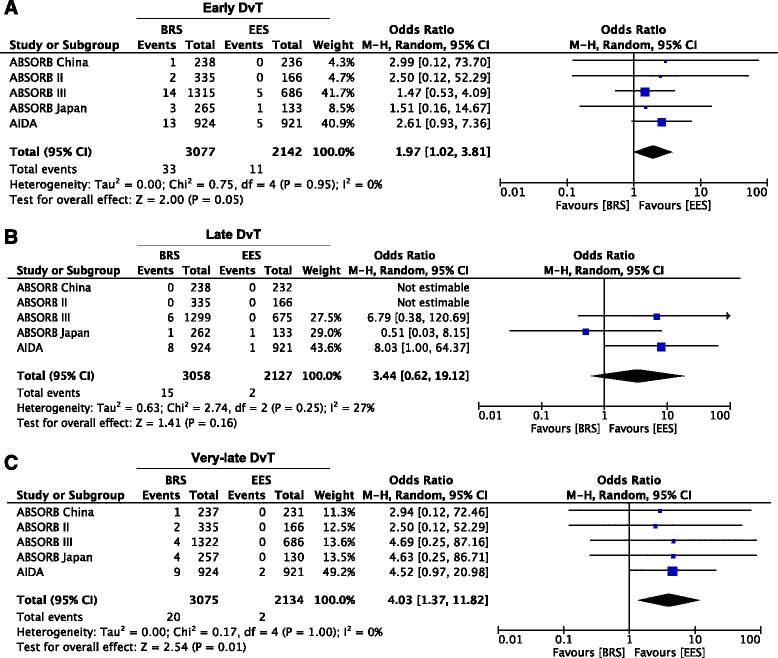
Meta-analysis of Early, Late and Very-late Scaffold Thrombosis. Panel **a**. Forest plot and summary effect of the difference in the incidence of Early DvT, showing a significantly lower incidence in the EES arm (*p* = 0.05). Panel **b**. Forest plot and summary effect of the difference in the incidence of Late DvT, showing no difference between BRS and EES. Panel **c**. Forest plot and summary effect of the difference in the incidence of Very-late DvT, showing a significantly lower incidence in the EES arm (*p* = 0.01)

BRS was associated with higher rates of DvT compared with EES (2.3% vs 0.7%; OR = 3.22; 95% CI 1.86 to 5.57; *p* < 0.0001, I^2^ = 0) (Fig. 4). Interestingly, the incidence of both early (within 30 days after implantation, 1.1% vs 0.5%, OR 1.97, 95% CI 1.02 to 3.81; *p* = 0.05) and very late DvT (>1 year, 0.6% vs 0.1%, OR 4.03, 95% CI 1.37 to 11.82; *p* = 0.01)) was higher with BRS compared with EES (Fig. 5a, c), with the majority of events occurring within 30 days (*n* = 44). Conversely, although numerically higher with BRS compared to EES, the incidence of late DvT (30 days to 1 year) was not statistically different between devices (0.5% vs 0.1%, OR 3.44%, 95% CI 0.62 to 19.12; *p* = 0.16) (Fig. 5b).

These results were unchanged when fixed effects model was used. There was no evidence of publication bias by visual inspection of Funnel plots and by Egger’s test (Additional file 2: Figure S2).

## Discussion

This is the most comprehensive and updated meta-analysis of randomized studies comparing the long-term outcome after treatment of coronary artery disease with everolimus-eluting BRS or the equivalent metallic stent (EES). Summing up the best clinical evidence available to date, including 5 randomized studies and 5219 patients, we found that: a) BRS was associated with higher rates of TLF compared with EES; b) BRS was associated with higher rates of TV-MI compared with EES; c) BRS was associated with higher rates of DvT compared with EES; d) very-late DvT was higher with BRS compared with EES.

These results are not surprising, as similar trends were reported both in randomized and observational studies [20, 21]. In particular, a previously published patient-level meta-analysis reported a higher incidence of device thrombosis with the BRS, compared with the equivalent metallic EES [22]. However, their observation period was limited to 1 year and the studies were heterogeneous with regards to the enrollment of acute coronary syndrome and stable CAD patients [23]. Similarly, a recent published meta-analysis of RCTs and observational studies had already reported a higher risk for TLF and DvT in BRS-treated patients [24–26]. In this context, our results represent a robust confirmation of the association between use of BRS and a higher rate of DvT, and are the first report of differences in DvT early, late, and very-late after implantation.

The pathophysiology of scaffold thrombosis, and the possible explanations for the increased risk as compared to drug-eluting stents (DES), and particularly the role of the implantation technique [27, 28] have been previously investigated. The implantation technique was not homogeneous across studies and instructions for use were not systematically followed in all studies. In fact, appropriate sizing of the balloon to be used for pre-dilation was not warranted in all patients enrolled in the Amsterdam Investigator-Initiated Absorb Strategy All-Comers Trial (AIDA). More generally, the BRS-specific implantation protocol, prescribing long inflation times and systematic high-pressure post-dilation with a non-compliant balloon, was not homogeneously adopted in all included studies, despite it was strongly recommended to achieve optimal implantation results [29–31]. In addition, intravascular imaging guiding during BRS implantation has been reported to have a major positive impact on patients’ outcome but was not frequently used across the selected studies [32–34]. Thus, a potential explanation for the observed increased risk of DvT in the randomized trials may relate to suboptimal implantation techniques, rather than to the intrinsic properties of BRS. Findings of this meta-analysis have relevant potential implications. The concern raised about BRS thrombosis, together with the lack of a clear advantage in terms of clinical efficacy, potentially undermines the future development of this promising class of coronary devices. In fact, the added value of the so-called vascular restoration therapy is still waiting for a proof of evidence, while interventional cardiologists have largely experienced the technical challenges in implanting the device, including worse trackability than best-in-class equivalent EES, longer procedural times, larger amounts of contrast medium necessary for successful implantation.

Collectively, the data emphasize the importance of appropriate lesion selection and accurate application of proper implantation technique. As well, a new generation of BRS should warrant a better radial strength, a sleeker endoluminal profile, a smaller footprint, and resorption processes that do not interact with the vessel wall.

### Study limitations

Although no large heterogeneity was found between the randomized studies included in the present analysis, it entails possible limitations of the original studies included. There were differences in the study design, patients’ and procedural characteristics. To account for these potential sources of heterogeneity, we used a random effects model for all analyses. Even though the present analysis only included high quality, randomized studies, some potential source for bias may still persist. Unfortunately, a patient level meta-analysis couldn’t be possible because of lack of data. Additionally, in two of the trials, 2-year results had been presented but not have not been published yet [15, 19]. In addition, since angina recurrence was not systematically reported in the original studies, we could not analyze the impact of BRS on this specific outcome. Moreover, we only focused on the Absorb, as this was the only type of BRS with several randomized studies and reporting long-term outcomes. Hence, our findings may not entirely apply to other BRS platforms. As well, the assessment of publication bias is limited by the small number of trials included, preventing a definitive exclusion of potential small study effect. Finally, further data on the impact of a BRS-specific implantation technique and the role of sizing on DvT are still needed, to confirm that BRS can deliver the same results as DES with the appropriate implantation techniques [35].

## Conclusions

BRS may be associated with worse two-years clinical outcomes compared with EES in patients with CAD. In particular, the current data expand previous observations of an increased risk of early DvT to long-term follow-up periods.

## Additional files

Additional file 1: Figure S1. Risk of bias. Summary of the study quality analysis. (PPTX 80 kb)

Additional file 2: Figure S2. Funnel plots. Funnel plots for TLF, TLR, TV-MI, Cardiac Death, Definite/Probable DvT, Early, Late and Very-late DvT, demonstrating no evidence of publication bias. Each circle represents a study. Study precision (reported on the y-axis as the Standard Error of the Log OR) is plotted against the summary effect. (PPTX 158 kb)

## Abbreviations

AIDA: Amsterdam investigator-initiated absorb strategy all-comers trial
BRS: Bioresorbable scaffolds
CI: Confidence interval
DES: Drug eluting stent
DvT: Device thrombosis
EES: Everolimus eluting stent
ID: Ischemia-driven
MI: Myocardial infarction
OR: Odds ratio
PCI: Percutaneous coronary intervention
RCT: Randomized controlled trial
TLF: Target lesion failure
TLR: Target lesion revascularization
