# Supplementary material for: Long-term outcome of bioresorbable vascular scaffolds for the treatment of coronary artery disease: a meta-analysis of RCTs
Source: BMC Cardiovasc Disord. 2017 Jun 7;17:147. doi: 10.1186/s12872-017-0586-2 (PMC5463321; doi:10.1186/s12872-017-0586-2)
Supplement: Supplementary file 1 — Risk of bias. Summary of the study quality analysis. (PPTX 80 kb) [file 12872_2017_586_MOESM1_ESM.pptx]

## Slide 1
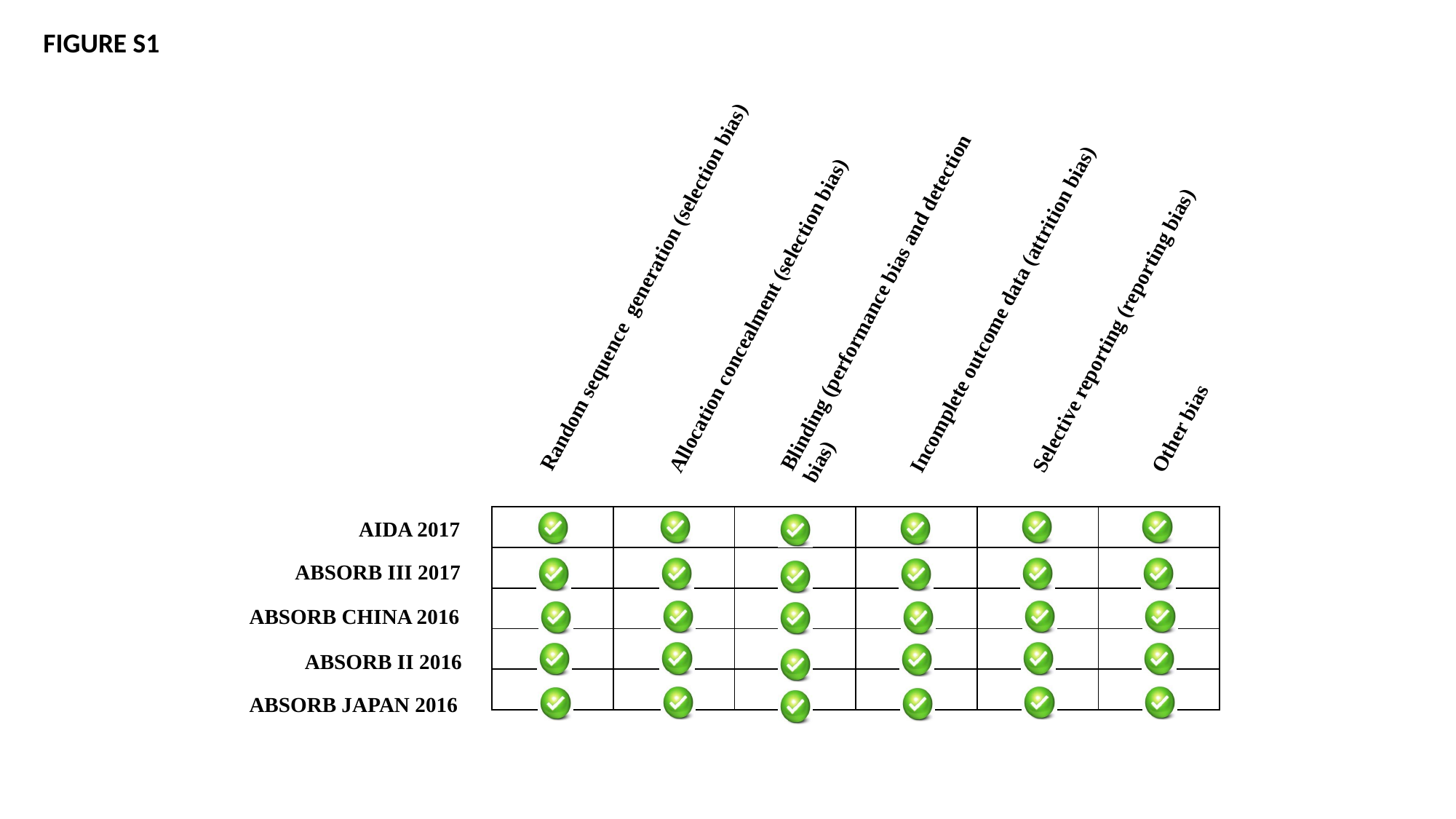

FIGURE S1
Blinding (performance bias and detection bias)
Random sequence generation (selection bias)
Allocation concealment (selection bias)
Incomplete outcome data (attrition bias)
Selective reporting (reporting bias)
Other bias
| | | | | | |
| --- | --- | --- | --- | --- | --- |
| | | | | | |
| | | | | | |
| | | | | | |
| | | | | | |
 AIDA 2017
ABSORB III 2017
ABSORB CHINA 2016
 ABSORB II 2016
ABSORB JAPAN 2016
