# Supplementary figures and images for: Long-term outcome of bioresorbable vascular scaffolds for the treatment of coronary artery disease: a meta-analysis of RCTs
Source: BMC Cardiovasc Disord. 2017 Jun 7;17:147. doi: 10.1186/s12872-017-0586-2 (PMC5463321; doi:10.1186/s12872-017-0586-2)

## Slide 1
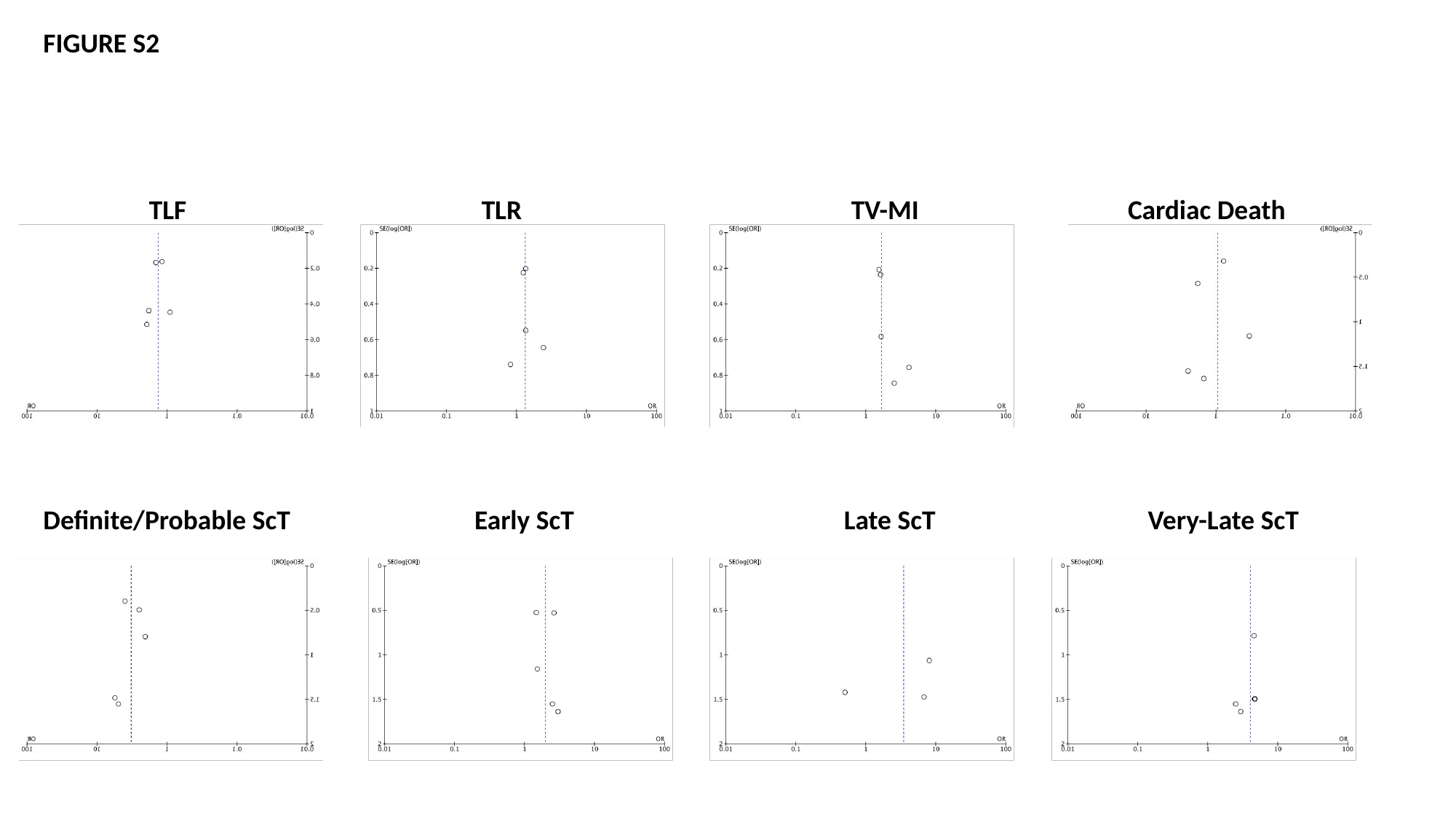

FIGURE S2
TLF
TLR
TV-MI
Cardiac Death
Definite/Probable ScT
Early ScT
Late ScT
Very-Late ScT

Supplement: Supplementary file 2 — Funnel plots. Funnel plots for TLF, TLR, TV-MI, Cardiac Death, Definite/Probable DvT, Early, Late and Very-late DvT, demonstrating no evidence of publication bias. Each circle represents a study. Study precision (reported on the y-axis as the Standard Error of the Log OR) is plotted against the summary effect. (PPTX 158 kb) [file 12872_2017_586_MOESM2_ESM.pptx]
